# MR-Imaging of Meniscal Substitution

**DOI:** 10.5334/jbr-btr.1168

**Published:** 2016-11-19

**Authors:** Tineke De Coninck, Peter Verdonk, Koenraad Verstraete

**Affiliations:** 1 Department of Radiology, Ghent University Hospital, De Pintelaan 185, B-9000 Ghent, Belgium; 2 Antwerp Orthopedic Center, Monica Hospitals, Harmoniestraat 68, 2018 Antwerp, Belgium

**Keywords:** Meniscus, MRI, Knee, Implants, Allografts

## Abstract

More than a century ago, the menisci were considered to be the functionless remains of a leg muscle. Gradually the usefulness and function of the meniscus was investigated and proven, and the link between total meniscectomy, radiographic osteoarthritis and reduced knee function was made. Subsequently, partial meniscectomy was introduced in the clinical practice. However, the frequency of symptomatic knee osteoarthritis was not substantially lowered. Therefore, meniscal repair was introduced for younger individuals with traumatic meniscus lesions with a good healing potential. Later on in the development process, the quest for meniscal replacement strategies arose. The introduction of allogenic, xenogenic and artificial materials followed in research and clinical settings. Nowadays, a lot of research is conducted on meniscal substitutes, because meniscal injuries are a very common problem in the general population. The imaging of the meniscus is running parallel to this evolution. With the development of magnetic resonance imaging (MRI), the meniscus could be perfectly visualized. A lot of studies were published on imaging of the normal meniscus, and subsequently meniscal pathology on MRI was investigated. In the current literature, a growing number of papers describe the MRI findings in artificial meniscus replacements.

## Meniscal Pathology

Meniscal damage causing knee pain and disability is a frequently encountered problem by orthopaedic surgeons [1,2,3,4]. Injuries occur more frequently in the medial meniscus (approximate ratio medial:lateral = 2:1), particularly in the stable knee or in the chronic anterior cruciate ligament (ACL)-deficient knee [5], possibly due to the more stable fixation of the medial meniscus. Lateral meniscal tears are more common in association with an acute ACL tear [6].

Different tears commonly arise from different origins; traumatic tears are often associated with a known insult to the knee and may be isolated or associated with ligament or articular surface injury. Traumatic tears generally occur in younger, active individuals [7]. Cumulative stress on the meniscus may result in degenerative tears with a horizontal or radial pattern and can correlate with the presence of associated chondromalacia [7]. Degenerative tears in older patients more often tend to be complex, consisting of a combination of the above-mentioned tears and displaying largely in the posterior horn. A specific type of radial tear up to 9 mm from the root attachment can be considered a true ‘root tear’. These root tears significantly alter the native biomechanics of the posterior meniscal root [8].

Magnetic resonance imaging (MRI) may miss approximately one-third of radial tears adjacent to the posterior root attachment of the medial meniscus [9]. A recent MRI study also demonstrated that posterior lateral meniscal root tears often have intact meniscofemoral ligaments that prevent meniscal radial displacement [10]. Radial displacement of the meniscus has been defined as displacement of the meniscus with respect to the outer margin of the tibial plateau and is considered pathological when exceeding 3 mm. This displacement occurs due to tears at the base of the meniscus, radial tears, complex tears, meniscal degeneration and degenerative joint disease. Because of this displacement, the meniscus can no longer redistribute forces throughout the knee joint. More stress is transmitted to the femorotibial cartilage, leading to cartilage degeneration, flattening of the femoral condyles and osteophyte formation [11,12,13]. Therefore, it is becoming increasingly recognized that meniscal root tears often require repair with the attempt to restore the native structure and function of the root attachment [8].

## Treatment

### Meniscal repair

Only about 10% of meniscal tears are amenable to meniscal repair due to the limited vascular supply of the meniscus. Three criteria have to be considered when choosing for meniscal repair. The first and foremost criterion is the stability of the tear [14]. Any tear with a fragment that is displaced more than 3 mm on MRI is considered unstable. Complex tears and full-thickness vertical tears longer than 1 cm are also considered unstable. A second criterion for suturing of a meniscal tear is an intact inner fragment. The third prerequisite that needs to be considered is the location of the tear. Peripheral tears through or within 4 mm from the meniscosynovial junction are a good indication for suturing [15]. Overall, meniscal repair is most often performed for vertical longitudinal tears. Especially in radial root tears, meniscal repair has been announced for its ability to restore femorotibial joint loading profiles to a more native pattern, thereby preventing meniscal radial displacement and the rapid progression of osteoarthritis. Therefore, it is becoming increasingly recognized that meniscal root tears often require repair that attempts to restore the native structure and function of the meniscal root attachments.

### (Partial) meniscectomy

The most commonly performed treatment option for meniscal tears is resection of meniscal tissue via partial meniscectomy, currently mostly performed arthroscopically [16]. A total meniscectomy is only prescribed in the case of complex meniscal tears or when no healthy tissue remains [17].

Some patients develop a post-meniscectomy syndrome on the short-term after surgery: they have pain and swelling in the index knee and are prone to develop early osteoarthritis. To treat this group of patients, meniscal substitution mechanisms were developed.

## Meniscal substitution

### Complete substitution

#### A. Meniscus Allografts

The primary indication for meniscal transplantation are people younger than 50 years of age who previously have had a total meniscectomy and complain of pain in the involved compartment. A second group benefiting from meniscal transplantation are patients with a defect ACL who previously have had a medial meniscectomy [18]. A third group are young patients who need a total meniscectomy. They are eligible for transplantation to postpone or prevent early joint degeneration [19,20].

Currently, no level I evidence exists to support the role of a meniscus transplant in halting the progression of osteoarthritis [21].

**Figure 1:**
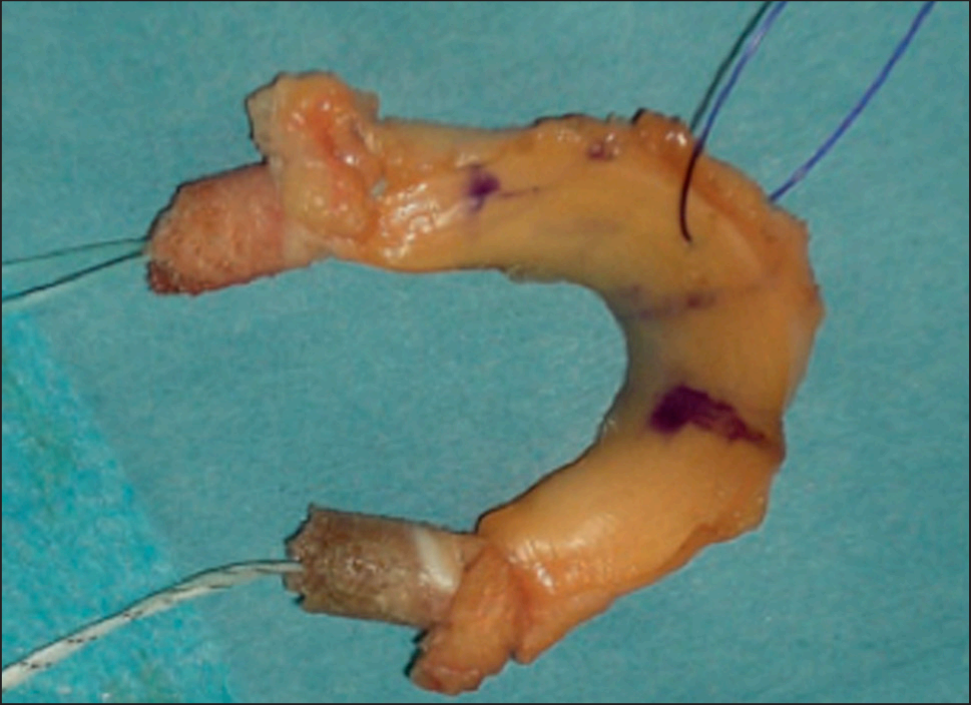
Lateral meniscal allograft prepared for arthroscopic implantation (2).

Three types of allografts are available according to the preservation method: fresh, deep frozen (fresh-frozen) and cryopreserved menisci [22,23]. Accurate graft sizing is essential for survival and to maximize the cartilage-protecting effect in the acceptor’s knee. A standard radiography combined with the technique of Pollard is still the standard technique for graft sizing. Hereby the width of the meniscus is determined anteroposteriorly. The length of the meniscus is measured by multiplying the length of the tibial plateau on a lateral view by 0.8 for the medial meniscus and by 0.7 for the lateral meniscus. A size tolerance of 5% is accepted. Most commonly, the periphery of the allograft is sutured to the remaining peripheral rim or the joint capsule. The meniscal horns are fixed with either small, attached bone plugs or soft tissue fixation with sutures [24,25]. In both methods the goal is a firm anchoring between the anterior and posterior horn (Figure 1).

#### B. Total medial meniscus implant

**Figure 2:**
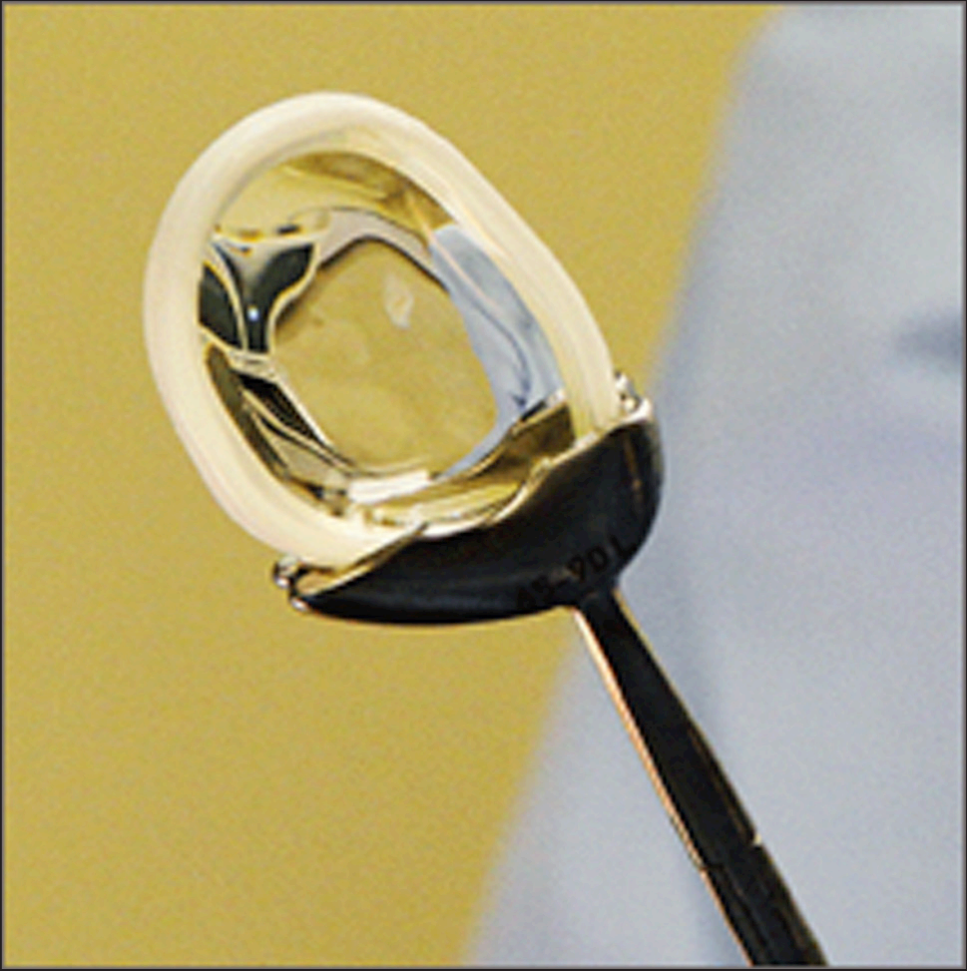
The medial meniscal implant composed of a polycarbonate-urethane matrix, reinforced with circumferential polyethylene fibres (Courtesy of Active Implants Corp., Memphis, TN, USA).

The successful use of biological solutions, such as meniscal allografts and biodegradable scaffolds, is usually limited to patients below 50 years of age. Thus, in order to fulfil the need for treatment of chronic, middle-aged patients with a dysfunctional and painful meniscus, a synthetic and functional free-floating polyethylene reinforced polycarbonate urethane (PCU) meniscus implant (NUsurface®, Active Implants Corp., Memphis, TN, USA) was developed for medial meniscal replacement [26,27] (Figure 2). The first clinical results have not been published yet.

This PCU-device is implanted during arthroscopy. The native meniscus is resected up until the stable meniscal wall and the resultant defect is measured. An implant with the appropriate size is placed into the joint space, by performing a mini-arthrotomy. The implant is positioned within the pocket-like compartment created for all patients, namely the medial femorotibial compartment.

### Partial meniscus substitution

**Figure 3:**
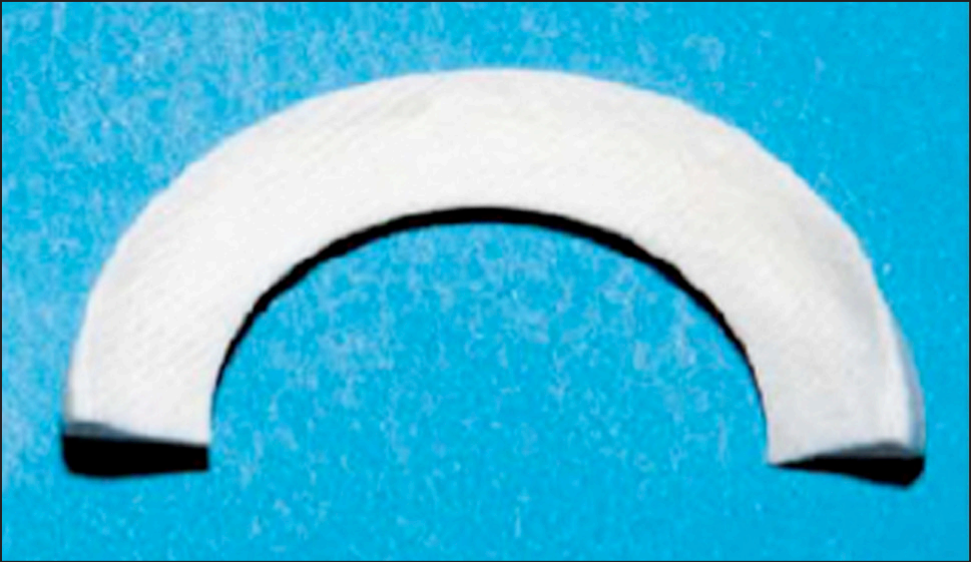
Medial collagen meniscus implant (Courtesy of Ivy Sports Med Patient Brochure).

The irreparable lesions in the avascular zone of the meniscus are treated with partial meniscectomy. In the short-term, this procedure leads to a favourable clinical outcome, but on the long-term, irreparable damage and osteoarthritis appear. A lot of research has been performed during the last decade, searching for materials that can replace the damaged part of the meniscus, with purpose to prevent or slow down cartilage damage and to diminish pain. There are currently two commercially available scaffold options for partial meniscal substitution outside the United States and in Europe: the Collagen Meniscus Implant (CMI®, Ivy Sports Medicine, Gräfelfing, Germany) and the Actifit® (Orteq Bioengineering, London, UK) (Figure 3). The indications for partial meniscus substitution are restricted to adult patients with the following profile: patients with post-meniscectomy symptoms, chondral injuries up to grade 2 according to International Cartilage Repair Society (ICRS)-criteria, stable knees or knees stabilized in the same procedure and a preserved meniscal rim [28].

The CMI® is a collagen matrix designed to induce vascular ingrowth. It is bioresorbable, with most of the scaffold resorbed over 12–18 months [29,30] (Figure 3).

The other commercially available partial meniscus implant is the Actifit®, a biodegradable highly porous scaffold made from aliphatic polyurethane (PU). The implant composes of a three-dimensional matrix with pores that are mutually connected, with the goal of these pores to allow the ingrowth of vessels when they are connected with the vascularized part of the native meniscus [31]. The scaffold is arthroscopically implanted after performing a partial meniscectomy, and it is sutured to the native peripheral meniscal rim with 4 to 5 sutures [32]. A case-series study in 52 human patients demonstrated tissue ingrowth and vascular perfusion on dynamic contrast-enhanced MRI 3 months after implantation in 35 of 43 patients. Patients with irreparable medial and lateral meniscal defects showed a statistically significant improvement in pain and activity scores 6 months after implantation of the Actifit® scaffold compared to baseline. Two-year results of the same series of 52 patients showed statistically significant improvements from baseline in all clinical outcomes examined [28]. Greater than 90% of patients demonstrated improved ICRS articular cartilage scores on MRI at 24 months and the number of adverse events and serious adverse events were comparable to partial meniscectomy [28].

To summarize, the available implants for partial meniscal defects seem safe. For acute irreparable meniscal lesions, the additional value of the CMI® could not be proven. The implants seem to improve the clinical scores in chronic lesions compared to the preoperative situation, but whether the implanted knee joint functions better than meniscectomized control knees is still doubtful. A prospective randomized study including an Actifit® implant group and a partial meniscectomy group is necessary to assess the added value of this meniscal substitute. Moreover, extended trial periods should demonstrate the long-term effects of treatment with the Actifit® implant (29).

## MR-imaging of Meniscal Substitutes

### MRI technique of the postoperative knee

A screening examination is preferentially performed consisting of paired fast spin-echo (FSE) proton density (PD) and fat-suppressed T2-weighted MR images in all planes. If substantial postoperative metallic artefacts are present, a metal-artefact reduction protocol can be performed including optimized FSE PD and FSE inversion recovery pulse sequences. MRI at 3T provides a higher resolution and thinner slices, which is an advantage in postoperative imaging. However, images are more prone to susceptibility and chemical shift artefacts [33,34].

The accuracy of detecting residual or recurrent tears after meniscectomy does not increase significantly by performing a direct arthrography instead of an indirect MR arthrography or conventional MRI [35,25].

Even in the postoperative phase, the accuracy of detecting residual or recurrent tears after meniscectomy does not increase significantly by performing a direct arthrography instead of an indirect MR arthrography or conventional MRI [25,33,35,36].

Arthroscopic trajectories can be recognized on MRI by the presence of small metallic artefacts caused by microscopic metal fragments along the instrumentation tracts. On spin-echo (SE) and FSE MRI, these fragments cause tiny areas of signal loss. On gradient-echo (GRE) sequences, which are much more sensitive to differences in magnetic susceptibility, these artefacts are much more obvious [35].

Fibrous scar tissue formation along the course of the arthroscopic tract can be recognized as areas of linear relative low signal on all sequences. In the first few months after surgery, however, early granulation tissue demonstrates high signal intensity on T2-weighted images.

If during surgery the patellar retinaculae or the patellar tendon have been pierced by arthroscopic instruments, they can demonstrate a permanent fibrous thickening. Tiny metallic fragments can also become embedded within the knee joint cartilage, especially in the posterior part of the femoral condyles [35].

### MRI of the allograft transplant meniscus

MRI is the golden standard for non-invasive imaging of meniscal allografts. Comparison between MR findings and second-look arthroscopy demonstrates that MRI is an accurate indicator of the status of the graft with regard to its position within the femorotibial joint, the capsular attachment, the detection of areas of meniscal transplant degeneration, and the condition of the adjacent articular cartilage [35, 37, 38].

**Figure 4:**
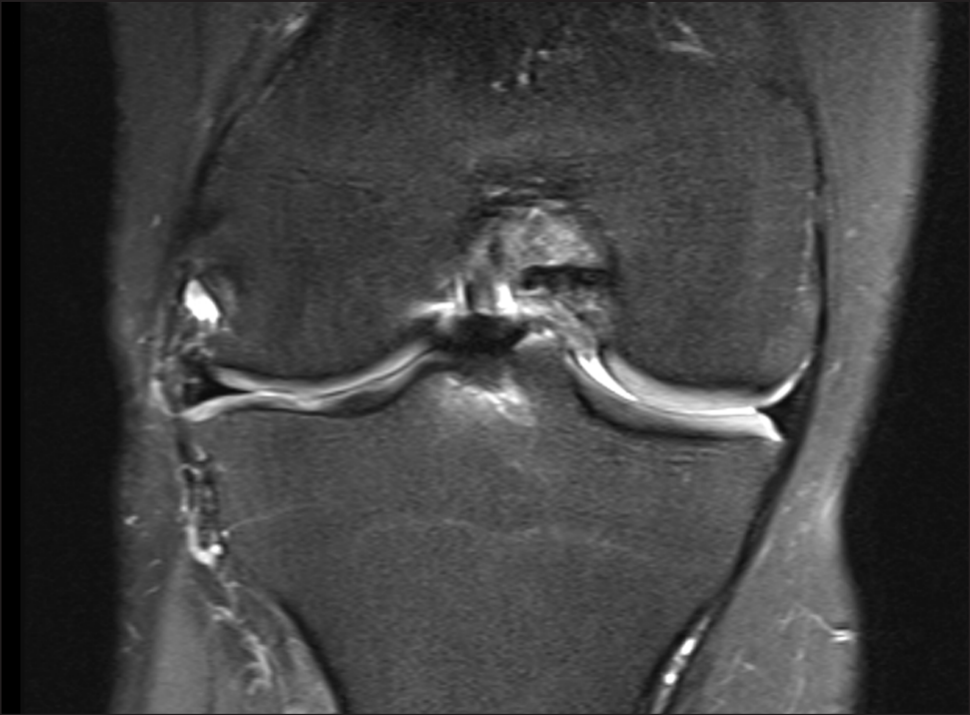
Coronal fat saturation T2-weighted fast spin echo MR-image of a meniscus allograft in the lateral compartment. Radial displacement of the allograft is a quite common finding after meniscal allograft transplantation.

Radial displacement of the body of the meniscus, to some degree, is a quite common finding after meniscal allograft transplantation [39,40,41] (Figure 4). It can vary from slight displacement to dislocation of portions of the allograft into the peripheral gutters. Some authors consider the radial displacement as a first sign of subsequent joint degeneration [11,42]. However, Verdonk et al. did not find a statistically significant difference in progression of cartilage degeneration between those with and those without radial displacement over more than a 10-year period [20]. Lee et al. observed that the amount and incidence of meniscal allograft radial displacement on MRI was greater after medial than lateral allograft transplantation, in both the coronal and sagittal planes. The radial displacement, however, did not correlate with any clinical outcome [43]. A discrepancy has been observed, however, between morphological MR findings and subjective parameters. Radial displacement has been correlated significantly with osteoarthritis progression, but not clinical outcome, by follow-up at four years [44].

Radial displacement of arthroscopically inserted grafts is significantly less than the radial displacement of grafts inserted with open soft-tissue fixation on MRI. In all cases, both open and arthroscopic, the RD of allografts was significantly larger than that of normal menisci [45].

**Figure 5:**
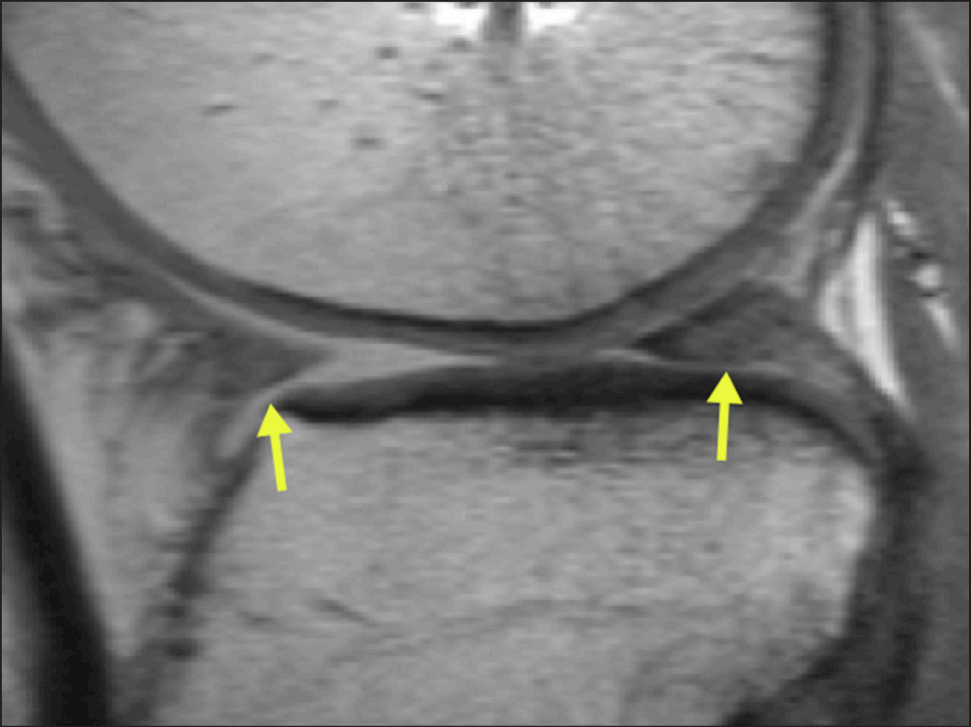
Sagittal T1-weighted fast spin echo MR-image of a lateral meniscal allograft. The allograft demonstrates generalized high signal (yellow arrow). These signal changes are the result of a change in water content and do not necessarily have to be a sign of degeneration.

The transplanted meniscus preferably demonstrates the same signal intensity as the native meniscus. However, generalized or focal areas of grade 3 high signal are often seen in grafts [46] (Figure 5). These signal changes can already appear shortly after surgery and can remain unchanged at follow-up, or they may progress to at least one year postoperatively, which is more frequently seen in the lateral compartment. Verdonk et al. hypothesized that these signal changes do not represent tears but rather changes in water content and extracellular matrix composition and absence of a dense collagen fibre network. Also decreased width and increased thickness of the body segment can be observed [20].

**Figure 6:**
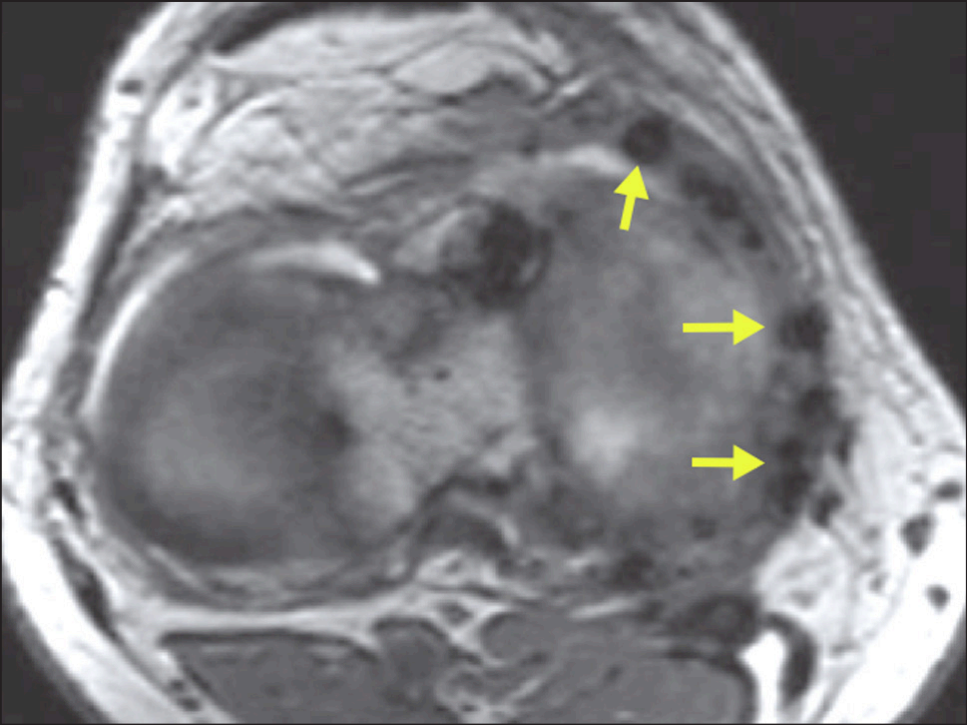
Axial T1-weighted fast spin echo MR-image of a medial meniscal allograft. The whole length of the meniscocapsular junction is obscured by multiple micrometallic artefacts caused by surgical manipulation.

The peripheral capsular attachment also demonstrates high signal intensity that corresponds histologically to scar tissue with cellular ingrowth and revascularization. However, the whole length of the meniscocapsular junction is more often obscured by multiple micrometallic artefacts caused by surgical manipulation (Figure 6). This feature can allow the radiologist to identify the meniscus as an allograft, even if the shape, position, and signal intensity are normal (35).

### MRI of the artificial meniscus

**Figure 7:**
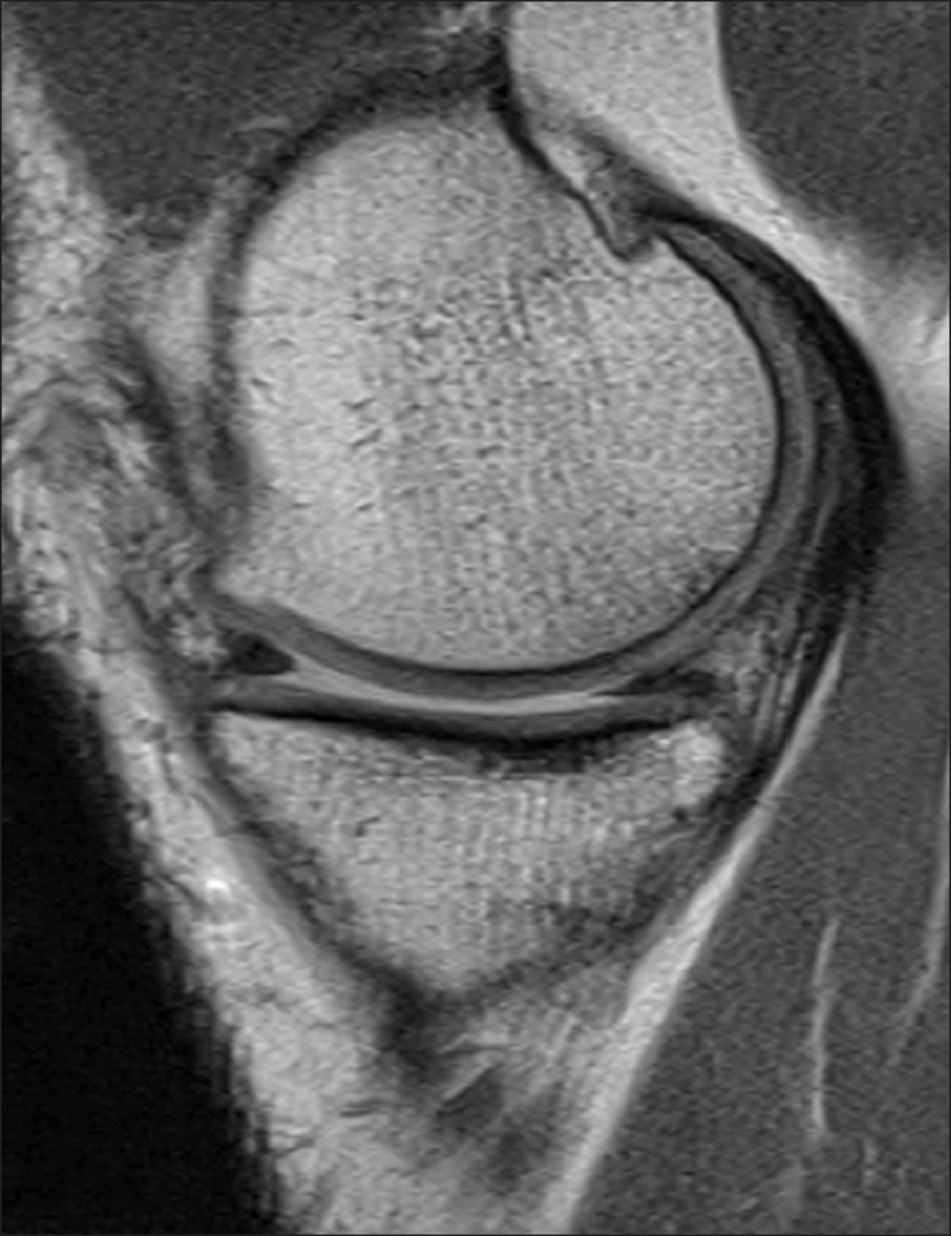
Sagittal T1-weighted fast spin echo MR-image demonstrating a small but intact collagen meniscus implant at the posterior horn of the medial meniscus.

Only a few number of reports have been published on the findings of in-vivo artificial meniscus implants on MRI [46,47,48] and even fewer authors have reported on the long-term outcomes [25,49]. Shrinkage or tissue loss has been reported in collagen menisci (Figure 7). A report on the PU-scaffold mentions no shrinkage but the appearance of focal defects in the posterior horn [35].

**Figure 8:**
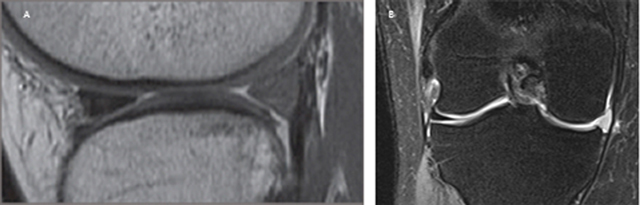
(A) Sagittal T1-weighted fast spin echo MR-image demonstrating the slightly hyperintense polyurethane scaffold at the posterior horn of the lateral meniscus. **(B)** Coronal fat saturation T2-weighted fast spin echo MR-image demonstrating a hyperintense polyurethane scaffold in the medial compartment. The native meniscal rim can be seen as a black rim encapsulating the scaffold. The scaffold demonstrates moderate radial displacement.

On MRI the signal intensity of both the collagen and PU-scaffold is markedly higher on T2-weighted images and moderately higher on T1-weighted images, presumably because of the highly porous structure and water content and absence of a collagen fibre network (Figure 8A–B). This hyperintense signal tends to diminish over the years as the scaffold matures and with ingrowth of cells and collagen network creation, but never reaches the same low signal of the normal fibrocartilaginous meniscus [35].

A two-year follow-up study on the polyurethane based scaffold (Actifit®) demonstrated in 52 patients on MRI a stable or improved ICRS articular cartilage score in 93% of patients between baseline and two-year follow-up [50].

Another two-year follow-up study reported that limited medial meniscal radial displacement was present preoperatively but increased by 2 mm after scaffold implantation (Figure 8B). Lateral radial displacement was also present preoperatively but did not increase after scaffold implantation. A strong negative correlation was found between the rim and postoperative medial radial displacement; a thicker rim limited radial displacement because peripheral longitudinal fibres were still present and retained hoop stress. However, in the lateral compartment, rim thickness did not correlate with radial displacement because this displacement was already strongly present preoperatively. Finally, no correlations were observed between scaffold radial displacement and clinical outcome scores, either preoperatively or postoperatively [51].

The collagen-based scaffold (Menaflex®) has a longer performance record, with initial three-year follow-up data already published in the mid-1990s [47]. With implant maturation, the signal intensity tended to decrease over time, although myxoid degeneration was present in most implants.

In a subsequent study of 25 patients with a minimum follow-up of 10 years, a normal decrease in size of the implant over time was observed on MRI, but joint-space narrowing was minimal or absent [52].

In a recent study of 76 patients followed for 12 months postoperatively, the implant usually became partially resorbed (92%), appeared slightly hyperintense (90%), and was extruded by more than 3 mm (72%) [53].

**Figure 9:**
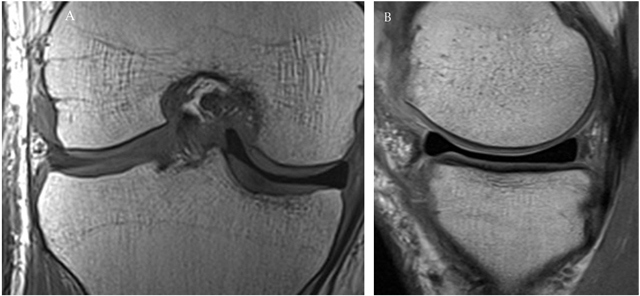
Total medial meniscus implant. Coronal **(A)** and sagittal **(B)** T1-weighted fast spin echo MR-images demonstrating the discoid medial meniscus implant with homogenous low signal intensity.

The total medial meniscus implant (NUsurface®) has a discoid shape on MRI. It demonstrates low signal intensity on both T1- and T2-weighted MR-images. The native meniscal rim can be identified as a rim that encapsulates the implant (Figure 9A–B) been reported in 118 patients, with follow-up MRI at six weeks and one year postoperative in 10 patients [54,55]. At six weeks, MRI showed medial compartment bone marrow oedema (9 of 10 patients) and medial pericapsular oedema (7 of 10 patients). At one year these findings had essentially resolved (except for one patient with residual bone marrow oedema) and did not correlate with pain scores or altered range of motion [55].

In a pilot study, the static kinematic behaviour of the implant was compared to the natural medial meniscus of the non-operated knee. No significant difference in the static kinematic behaviour between the implant and the non-operated knees was observed, which could suggest that the knee joint maintains its static kinematic properties after implantation. Secondly, the motion pattern, the radial displacement and the deformation of the meniscal implant were investigated in an open MR scanner. Radial displacement and meniscal height were not different, but anteroposterior movement was slightly different between the implant and the normal meniscus [56].

## Conclusion

During the last decades, several treatment options have been developed to treat meniscal injuries. A partial meniscectomy is still considered the gold standard to treat a meniscal tear. Meniscal repair is reserved for only a minority of patients with meniscal injuries. For patients suffering from a post-meniscectomy syndrome, meniscal substitution mechanisms have been developed. Meniscal allograft transplants and a polycarbonate-urethane implant (NUsurface®) can completely substitute the meniscus. Two mechanisms are available for partial meniscal substitution (Actifit® and CMI®). MRI is an excellent modality for the imaging of meniscus replacement strategies. Meniscal radial displacement of the implant is often observed on MRI, and is considered a class effect of meniscal substitution.
